# Bis(μ-2,2′-oxydibenzoato-κ^4^ *O*,*O*′:*O*′′,*O*′′′)bis­[(4,4′-dimethyl-2,2′-bipyridine-κ^2^ *N*,*N*′)zinc(II)] dihydrate

**DOI:** 10.1107/S1600536808010271

**Published:** 2008-04-23

**Authors:** Hong Cui, Wen-Juan Li, Ruo-Jie Tao

**Affiliations:** a Institute of Molecular and Crystal Engineering, College of Chemistry and Chemical Engineering, Henan University, Kaifeng 475001, Henan, People’s Republic of China; b Department of Civil and Environmental Engineering, East China Institute of Technology, 56 Xuefu Road, Fuzhou 344000, Jiangxi, People’s Republic of China

## Abstract

In the title compound, [Zn_2_(C_14_H_8_O_5_)_2_(C_12_H_12_N_2_)_2_]·2H_2_O, the Zn^II^ atom exhibits a distorted octa­hedral coordination geometry, defined by two N atoms from one 4,4′-dimethyl-2,2′-bipyridine ligand and four O atoms from two bridging 2,2′-oxydibenzoate ligands. The mol­ecule is a centrosymmetric dimer. π–π Stacking inter­actions are observed between the 4,4′-dimethyl-2,2′-bipyridine ligands, with a centroid–centroid distance of 3.649 (2) Å.

## Related literature

For related literature, see: Allen *et al.* (1987 ▶); Zhang *et al.* (2008 ▶).
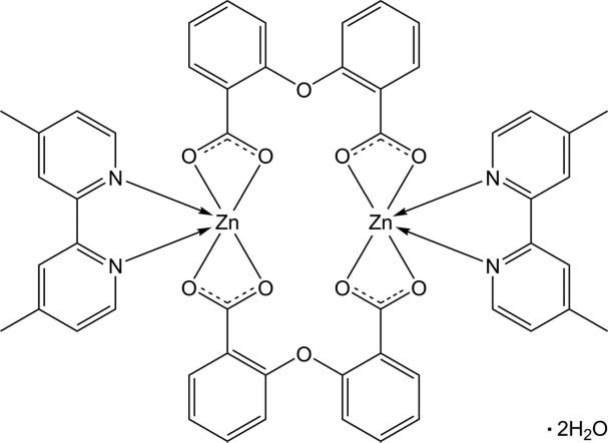


## Experimental

### 

#### Crystal data

[Zn_2_(C_14_H_8_O_5_)_2_(C_12_H_12_N_2_)_2_]·2H_2_O
*M* *_r_* = 1047.65
Triclinic, 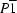

*a* = 10.425 (2) Å
*b* = 10.866 (2) Å
*c* = 11.960 (2) Å
α = 68.413 (4)°
β = 66.721 (3)°
γ = 78.348 (4)°
*V* = 1154.7 (4) Å^3^
*Z* = 1
Mo *K*α radiation
μ = 1.11 mm^−1^
*T* = 293 (2) K
0.20 × 0.16 × 0.15 mm

#### Data collection

Bruker SMART APEXII CCD area-detector diffractometer
Absorption correction: multi-scan (*SADABS*; Bruker, 2001 ▶) *T*_min_ = 0.808, *T*_max_ = 0.851
6797 measured reflections
4484 independent reflections
3788 reflections with *I* > 2σ(*I*)
*R*_int_ = 0.028

#### Refinement

*R*[*F* ^2^ > 2σ(*F* ^2^)] = 0.057
*wR*(*F* ^2^) = 0.123
*S* = 1.16
4484 reflections
318 parameters
H-atom parameters constrained
Δρ_max_ = 0.79 e Å^−3^
Δρ_min_ = −0.42 e Å^−3^

### 

Data collection: *APEX2* (Bruker, 2007 ▶); cell refinement: *SAINT* (Bruker, 2007 ▶); data reduction: *SAINT*; program(s) used to solve structure: *SHELXS97* (Sheldrick, 2008 ▶); program(s) used to refine structure: *SHELXL97* (Sheldrick, 2008 ▶); molecular graphics: *SHELXTL* (Sheldrick, 2008 ▶); software used to prepare material for publication: *SHELXTL*.

## Supplementary Material

Crystal structure: contains datablocks I, global. DOI: 10.1107/S1600536808010271/hy2127sup1.cif

Structure factors: contains datablocks I. DOI: 10.1107/S1600536808010271/hy2127Isup2.hkl

Additional supplementary materials:  crystallographic information; 3D view; checkCIF report

## Figures and Tables

**Table:** 

Zn1—O2	2.006 (3)
Zn1—O4^i^	2.053 (4)
Zn1—N2	2.059 (3)
Zn1—N1	2.086 (3)
Zn1—O5^i^	2.295 (5)
Zn1—O3	2.495 (4)

**Table:** 

O2—Zn1—O4^i^	100.39 (14)
O2—Zn1—N2	100.84 (13)
O4^i^—Zn1—N2	96.86 (14)
O2—Zn1—N1	106.07 (12)
O4^i^—Zn1—N1	153.52 (14)
N2—Zn1—N1	79.25 (11)
O2—Zn1—O5^i^	145.16 (14)
O4^i^—Zn1—O5^i^	57.76 (14)
N2—Zn1—O5^i^	108.05 (15)
N1—Zn1—O5^i^	98.21 (12)
O2—Zn1—O3	56.84 (11)
O4^i^—Zn1—O3	99.09 (14)
N2—Zn1—O3	154.58 (12)
N1—Zn1—O3	94.60 (12)
O5^i^—Zn1—O3	97.20 (14)

Symmetry code: (i) 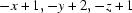.

**Table 2:** Hydrogen-bond geometry (Å, °)

*D*—H⋯*A*	*D*—H	H⋯*A*	*D*⋯*A*	*D*—H⋯*A*
O1*W*—H1*WA*⋯O3	0.85	2.04	2.845 (5)	158
O1*W*—H1*WB*⋯O5^i^	0.85	2.07	2.876 (6)	158

Symmetry code: (i) 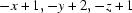.

**Fig. 1.**
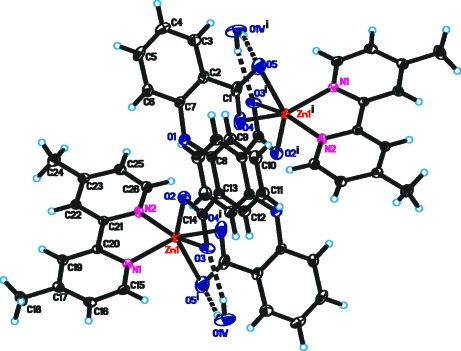
The molecular structure of the title compound. Displacement ellipsoids are drawn at the 20% probability level. Hydrogen bonds are indicated by dashed lines. [Symmetry code (i): 1 - x, 2 - y, 1 - z.]

**Fig. 2.**
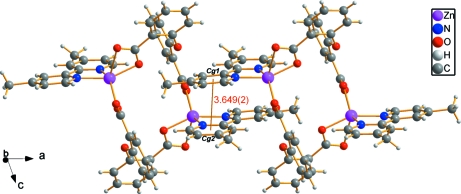
A view of the crystal packing, showing the π–π stacking interaction.

## supplementary crystallographic information

### Comment

As part of our ongoing studies (Zhang *et al.*, 2008), we synthesized the title compound and report here its crystal structure.

The bond lengths and angles of the title compound are within normal ranges (Allen *et al.*, 1987) (Table 1). Intermolecular hydrogen bonds are formed between the water molecule and the carboxylate groups (Fig. 1; Table 2). The molecule is centrosymmetric with an inversion center located at the midpoint of the Zn1 and Zn1^i^ atoms [symmetry code (i): 1 - *x*, 2 - *y*, 1 - *z*]. The asymmetric unit thus contains one-half molecule. The Zn^II^ atom exhibits a distorted octahedral coordination geometry, defined by two N atoms from one 4,4'-dimethyl-2,2'-bipyridine (dbpy) ligand and four O atoms from two 2,2'-oxydibenzoate (odb) ligands. The two carboxylate groups of each odb ligand coordinate to two different Zn atoms.

The π–π stacking interactions between the aromatic rings of the dbpy ligands are observed, with a centroid–centroid distance of 3.649 (2)Å [Fig. 2; *Cg*1 = the centroid of N1^ii^, C15^ii^, C16^ii^, C17^ii^, C19^ii^, C20^ii^ and *Cg*2 = the centroid of N2, C21, C22, C23, C25, C26; symmetry code: (ii) 2 - *x*, 2 - *y*, 1 - *z*].

### Experimental

The title compound was synthesized hydrothermally in a Teflon-lined autoclave (23 ml) by heating a mixture of H_2_odb (0.052 g, 0.2 mmol), dbpy (0.037 g, 0.2 mmol), Zn(NO_3_)_2_.6H_2_O (0.059 g, 0.2 mmol) and one drop of Et_3_N (pH = 8~9) in water (10 ml) at 393 K for 3 d. Colorless single crystals were collected in 56% yield based on Zn.

### Refinement

H atoms on C atoms were positioned geometrically and refined as riding atoms, with C—H = 0.93 (aromatic) and 0.96 (methyl) Å and with *U*_iso_(H) = 1.2(1.5 for methyl groups)*U*_eq_(C). The H atoms of the water molecule were located from a difference Fourier map and fixed in the final refinements with *U*_iso_(H) = 1.2*U*_eq_(O).

### Crystal data

**Table:**

[Zn_2_(C_14_H_8_O_5_)_2_(C_12_H_12_N_2_)_2_]·2H_2_O	*Z* = 1
*M**_r_* = 1047.65	*F*_000_ = 540
Triclinic, *P*1	*D*_x_ = 1.507 Mg m^−^^3^
Hall symbol: -P 1	Mo *K*α radiation λ = 0.71073 Å
*a* = 10.425 (2) Å	Cell parameters from 2731 reflections
*b* = 10.866 (2) Å	θ = 2.3–27.8º
*c* = 11.960 (2) Å	µ = 1.11 mm^−^^1^
α = 68.413 (4)º	*T* = 293 (2) K
β = 66.721 (3)º	Block, colorless
γ = 78.348 (4)º	0.20 × 0.16 × 0.15 mm
*V* = 1154.7 (4) Å^3^	

### Data collection

**Table:**

Bruker SMART APEXII CCD area-detector diffractometer	4484 independent reflections
Radiation source: fine-focus sealed tube	3788 reflections with *I* > 2σ(*I*)
Monochromator: graphite	*R*_int_ = 0.028
*T* = 293(2) K	θ_max_ = 26.0º
φ and ω scans	θ_min_ = 2.0º
Absorption correction: multi-scan(SADABS; Bruker, 2001)	*h* = −12→12
*T*_min_ = 0.809, *T*_max_ = 0.851	*k* = −13→13
6797 measured reflections	*l* = −10→14

### Refinement

**Table:**

Refinement on *F*^2^	Secondary atom site location: difference Fourier map
Least-squares matrix: full	Hydrogen site location: inferred from neighbouring sites
*R*[*F*^2^ > 2σ(*F*^2^)] = 0.057	H-atom parameters constrained
*wR*(*F*^2^) = 0.123	*w* = 1/[σ^2^(*F*_o_^2^) + (0.0391*P*)^2^ + 1.1151*P*] where *P* = (*F*_o_^2^ + 2*F*_c_^2^)/3
*S* = 1.16	(Δ/σ)_max_ = 0.001
4484 reflections	Δρ_max_ = 0.79 e Å^−^^3^
318 parameters	Δρ_min_ = −0.42 e Å^−^^3^
Primary atom site location: structure-invariant direct methods	Extinction correction: none

### Special details

**Table:**

Geometry. All e.s.d.'s (except the e.s.d. in the dihedral angle between two l.s. planes) are estimated using the full covariance matrix. The cell e.s.d.'s are taken into account individually in the estimation of e.s.d.'s in distances, angles and torsion angles; correlations between e.s.d.'s in cell parameters are only used when they are defined by crystal symmetry. An approximate (isotropic) treatment of cell e.s.d.'s is used for estimating e.s.d.'s involving l.s. planes.

### Fractional atomic coordinates and isotropic or equivalent isotropic displacement parameters (Å^2^)

**Table:**

	*x*	*y*	*z*	*U*_iso_*/*U*_eq_	
Zn1	0.69886 (4)	0.81936 (4)	0.62615 (4)	0.02963 (15)	
N1	0.8962 (3)	0.7397 (3)	0.6331 (3)	0.0290 (7)	
N2	0.7562 (3)	0.9615 (3)	0.6702 (3)	0.0294 (7)	
O1	0.7872 (3)	1.0671 (3)	0.1966 (3)	0.0402 (7)	
O2	0.7264 (3)	0.9119 (3)	0.4398 (3)	0.0476 (7)	
O3	0.7021 (4)	0.7032 (3)	0.4797 (3)	0.0653 (10)	
O4	0.5135 (3)	1.1471 (4)	0.2926 (4)	0.0723 (11)	
O5	0.4336 (4)	1.3253 (4)	0.1854 (5)	0.0931 (15)	
O1W	0.6185 (5)	0.4727 (3)	0.6971 (4)	0.0904 (14)	
H1WA	0.6406	0.5286	0.6219	0.109*	
H1WB	0.6035	0.5144	0.7494	0.109*	
C1	0.5330 (5)	1.2495 (5)	0.1998 (6)	0.0507 (12)	
C2	0.6804 (4)	1.2853 (4)	0.1109 (4)	0.0366 (9)	
C3	0.7046 (5)	1.4153 (4)	0.0292 (5)	0.0474 (11)	
H3	0.6285	1.4758	0.0235	0.057*	
C4	0.8373 (5)	1.4565 (4)	−0.0429 (5)	0.0522 (12)	
H4	0.8506	1.5438	−0.0965	0.063*	
C5	0.9501 (5)	1.3685 (5)	−0.0355 (4)	0.0497 (12)	
H5	1.0402	1.3968	−0.0825	0.060*	
C6	0.9309 (4)	1.2378 (4)	0.0413 (4)	0.0400 (10)	
H6	1.0078	1.1776	0.0441	0.048*	
C7	0.7965 (4)	1.1970 (4)	0.1141 (4)	0.0305 (8)	
C8	0.7349 (4)	0.9736 (3)	0.1771 (4)	0.0311 (8)	
C9	0.7182 (4)	0.9954 (4)	0.0613 (4)	0.0395 (10)	
H9	0.7370	1.0770	−0.0029	0.047*	
C10	0.6740 (5)	0.8971 (4)	0.0415 (4)	0.0469 (11)	
H10	0.6611	0.9133	−0.0354	0.056*	
C11	0.6487 (5)	0.7745 (4)	0.1344 (5)	0.0485 (11)	
H11	0.6212	0.7071	0.1200	0.058*	
C12	0.6650 (4)	0.7535 (4)	0.2488 (5)	0.0432 (10)	
H12	0.6476	0.6707	0.3113	0.052*	
C13	0.7062 (4)	0.8508 (4)	0.2750 (4)	0.0325 (8)	
C14	0.7124 (4)	0.8198 (4)	0.4060 (4)	0.0401 (10)	
C15	0.9622 (4)	0.6263 (3)	0.6140 (4)	0.0360 (9)	
H15	0.9129	0.5688	0.6071	0.043*	
C16	1.0999 (4)	0.5905 (4)	0.6042 (4)	0.0375 (9)	
H16	1.1412	0.5100	0.5917	0.045*	
C17	1.1763 (4)	0.6746 (4)	0.6130 (4)	0.0347 (9)	
C18	1.3281 (5)	0.6431 (5)	0.5984 (6)	0.0558 (13)	
H18A	1.3359	0.5799	0.6768	0.084*	
H18B	1.3699	0.7228	0.5796	0.084*	
H18C	1.3756	0.6065	0.5295	0.084*	
C19	1.1067 (4)	0.7924 (3)	0.6351 (4)	0.0311 (8)	
H19	1.1540	0.8513	0.6425	0.037*	
C20	0.9681 (4)	0.8214 (3)	0.6458 (3)	0.0260 (8)	
C21	0.8882 (4)	0.9448 (3)	0.6701 (3)	0.0258 (7)	
C22	0.9417 (4)	1.0362 (3)	0.6920 (3)	0.0285 (8)	
H22	1.0326	1.0222	0.6923	0.034*	
C23	0.8611 (4)	1.1487 (3)	0.7136 (4)	0.0329 (9)	
C24	0.9184 (5)	1.2489 (4)	0.7370 (5)	0.0450 (11)	
H24A	0.9678	1.2041	0.7953	0.068*	
H24B	0.8428	1.3055	0.7737	0.068*	
H24C	0.9812	1.3013	0.6572	0.068*	
C25	0.7257 (4)	1.1644 (4)	0.7143 (4)	0.0368 (9)	
H25	0.6680	1.2384	0.7288	0.044*	
C26	0.6775 (4)	1.0689 (4)	0.6931 (4)	0.0371 (9)	
H26	0.5860	1.0798	0.6949	0.045*	

### Atomic displacement parameters (Å^2^)

**Table:**

	*U*^11^	*U*^22^	*U*^33^	*U*^12^	*U*^13^	*U*^23^
Zn1	0.0253 (2)	0.0322 (2)	0.0321 (3)	−0.00478 (16)	−0.00982 (19)	−0.01000 (19)
N1	0.0305 (17)	0.0258 (15)	0.0303 (17)	−0.0043 (12)	−0.0116 (15)	−0.0061 (13)
N2	0.0243 (16)	0.0322 (15)	0.0351 (18)	−0.0003 (12)	−0.0112 (14)	−0.0144 (14)
O1	0.0490 (18)	0.0403 (15)	0.0358 (16)	−0.0131 (13)	−0.0210 (14)	−0.0053 (13)
O2	0.0485 (19)	0.0604 (19)	0.0336 (16)	−0.0092 (15)	−0.0156 (15)	−0.0106 (15)
O3	0.076 (2)	0.0526 (19)	0.053 (2)	−0.0105 (17)	−0.033 (2)	0.0133 (17)
O4	0.040 (2)	0.113 (3)	0.051 (2)	−0.034 (2)	−0.0021 (17)	−0.013 (2)
O5	0.040 (2)	0.069 (2)	0.155 (5)	0.0009 (18)	−0.023 (3)	−0.034 (3)
O1W	0.115 (4)	0.0360 (18)	0.091 (3)	0.000 (2)	−0.016 (3)	−0.014 (2)
C1	0.033 (3)	0.051 (3)	0.078 (4)	−0.005 (2)	−0.013 (3)	−0.038 (3)
C2	0.031 (2)	0.042 (2)	0.039 (2)	−0.0068 (17)	−0.0070 (19)	−0.0184 (19)
C3	0.051 (3)	0.038 (2)	0.053 (3)	−0.0012 (19)	−0.019 (2)	−0.015 (2)
C4	0.073 (4)	0.037 (2)	0.042 (3)	−0.021 (2)	−0.014 (3)	−0.006 (2)
C5	0.048 (3)	0.061 (3)	0.038 (2)	−0.032 (2)	−0.001 (2)	−0.015 (2)
C6	0.030 (2)	0.051 (2)	0.039 (2)	−0.0054 (18)	−0.0098 (19)	−0.016 (2)
C7	0.034 (2)	0.0351 (19)	0.0239 (19)	−0.0122 (16)	−0.0099 (17)	−0.0072 (16)
C8	0.0245 (19)	0.0331 (19)	0.035 (2)	−0.0020 (15)	−0.0083 (17)	−0.0120 (17)
C9	0.047 (3)	0.042 (2)	0.027 (2)	−0.0101 (19)	−0.012 (2)	−0.0077 (18)
C10	0.052 (3)	0.057 (3)	0.041 (3)	−0.004 (2)	−0.017 (2)	−0.025 (2)
C11	0.051 (3)	0.042 (2)	0.060 (3)	−0.003 (2)	−0.019 (2)	−0.026 (2)
C12	0.035 (2)	0.034 (2)	0.053 (3)	0.0008 (17)	−0.011 (2)	−0.012 (2)
C13	0.027 (2)	0.0325 (19)	0.034 (2)	0.0011 (15)	−0.0090 (18)	−0.0096 (17)
C14	0.024 (2)	0.050 (2)	0.034 (2)	−0.0015 (17)	−0.0089 (18)	−0.002 (2)
C15	0.043 (2)	0.0257 (18)	0.040 (2)	−0.0070 (16)	−0.015 (2)	−0.0086 (17)
C16	0.043 (2)	0.0263 (18)	0.042 (2)	0.0070 (16)	−0.016 (2)	−0.0138 (18)
C17	0.033 (2)	0.036 (2)	0.035 (2)	0.0031 (16)	−0.0127 (18)	−0.0122 (18)
C18	0.039 (3)	0.055 (3)	0.085 (4)	0.018 (2)	−0.031 (3)	−0.036 (3)
C19	0.032 (2)	0.0329 (19)	0.032 (2)	0.0001 (15)	−0.0148 (18)	−0.0115 (17)
C20	0.0282 (19)	0.0241 (16)	0.0236 (18)	−0.0021 (14)	−0.0077 (16)	−0.0068 (15)
C21	0.0258 (19)	0.0260 (17)	0.0230 (18)	−0.0020 (14)	−0.0065 (16)	−0.0071 (15)
C22	0.028 (2)	0.0301 (18)	0.0272 (19)	−0.0025 (15)	−0.0100 (17)	−0.0080 (16)
C23	0.039 (2)	0.0294 (18)	0.029 (2)	−0.0051 (16)	−0.0103 (18)	−0.0085 (17)
C24	0.052 (3)	0.038 (2)	0.053 (3)	−0.0059 (19)	−0.017 (2)	−0.023 (2)
C25	0.035 (2)	0.0309 (19)	0.044 (2)	0.0037 (16)	−0.013 (2)	−0.0149 (19)
C26	0.027 (2)	0.034 (2)	0.051 (3)	0.0043 (16)	−0.014 (2)	−0.0168 (19)

### Geometric parameters (Å, °)

**Table:**

Zn1—O2	2.006 (3)	C8—C13	1.405 (5)
Zn1—O4^i^	2.053 (4)	C9—C10	1.370 (6)
Zn1—N2	2.059 (3)	C9—H9	0.9300
Zn1—N1	2.086 (3)	C10—C11	1.379 (6)
Zn1—O5^i^	2.295 (5)	C10—H10	0.9300
Zn1—O3	2.495 (4)	C11—C12	1.375 (6)
N1—C15	1.335 (5)	C11—H11	0.9300
N1—C20	1.353 (4)	C12—C13	1.391 (5)
N2—C26	1.335 (5)	C12—H12	0.9300
N2—C21	1.350 (4)	C13—C14	1.501 (6)
O1—C8	1.372 (4)	C15—C16	1.378 (5)
O1—C7	1.389 (4)	C15—H15	0.9300
O2—C14	1.261 (5)	C16—C17	1.383 (5)
O3—C14	1.246 (5)	C16—H16	0.9300
O4—C1	1.231 (6)	C17—C19	1.399 (5)
O4—Zn1^i^	2.053 (4)	C17—C18	1.503 (5)
O5—C1	1.214 (5)	C18—H18A	0.9600
O5—Zn1^i^	2.295 (5)	C18—H18B	0.9600
O1W—H1WA	0.8500	C18—H18C	0.9600
O1W—H1WB	0.8500	C19—C20	1.380 (5)
C1—C2	1.512 (6)	C19—H19	0.9300
C1—Zn1^i^	2.519 (5)	C20—C21	1.489 (5)
C2—C7	1.389 (5)	C21—C22	1.378 (5)
C2—C3	1.395 (6)	C22—C23	1.383 (5)
C3—C4	1.372 (6)	C22—H22	0.9300
C3—H3	0.9300	C23—C25	1.385 (5)
C4—C5	1.367 (7)	C23—C24	1.497 (5)
C4—H4	0.9300	C24—H24A	0.9600
C5—C6	1.382 (6)	C24—H24B	0.9600
C5—H5	0.9300	C24—H24C	0.9600
C6—C7	1.386 (5)	C25—C26	1.377 (5)
C6—H6	0.9300	C25—H25	0.9300
C8—C9	1.392 (5)	C26—H26	0.9300
			
O2—Zn1—O4^i^	100.39 (14)	C10—C9—C8	120.3 (4)
O2—Zn1—N2	100.84 (13)	C10—C9—H9	119.8
O4^i^—Zn1—N2	96.86 (14)	C8—C9—H9	119.8
O2—Zn1—N1	106.07 (12)	C9—C10—C11	120.7 (4)
O4^i^—Zn1—N1	153.52 (14)	C9—C10—H10	119.7
N2—Zn1—N1	79.25 (11)	C11—C10—H10	119.7
O2—Zn1—O5^i^	145.16 (14)	C12—C11—C10	118.8 (4)
O4^i^—Zn1—O5^i^	57.76 (14)	C12—C11—H11	120.6
N2—Zn1—O5^i^	108.05 (15)	C10—C11—H11	120.6
N1—Zn1—O5^i^	98.21 (12)	C11—C12—C13	122.8 (4)
O2—Zn1—O3	56.84 (11)	C11—C12—H12	118.6
O4^i^—Zn1—O3	99.09 (14)	C13—C12—H12	118.6
N2—Zn1—O3	154.58 (12)	C12—C13—C8	117.0 (4)
N1—Zn1—O3	94.60 (12)	C12—C13—C14	118.4 (4)
O5^i^—Zn1—O3	97.20 (14)	C8—C13—C14	124.6 (4)
O2—Zn1—C1^i^	124.65 (15)	O3—C14—O2	121.3 (4)
O4^i^—Zn1—C1^i^	29.03 (14)	O3—C14—C13	119.1 (4)
N2—Zn1—C1^i^	104.76 (13)	O2—C14—C13	119.6 (3)
N1—Zn1—C1^i^	126.34 (15)	N1—C15—C16	123.2 (3)
O5^i^—Zn1—C1^i^	28.74 (14)	N1—C15—H15	118.4
O3—Zn1—C1^i^	98.76 (13)	C16—C15—H15	118.4
C15—N1—C20	117.9 (3)	C15—C16—C17	119.7 (3)
C15—N1—Zn1	127.7 (2)	C15—C16—H16	120.1
C20—N1—Zn1	114.0 (2)	C17—C16—H16	120.1
C26—N2—C21	118.4 (3)	C16—C17—C19	117.2 (3)
C26—N2—Zn1	126.0 (2)	C16—C17—C18	122.2 (4)
C21—N2—Zn1	115.5 (2)	C19—C17—C18	120.7 (4)
C8—O1—C7	121.0 (3)	C17—C18—H18A	109.5
C14—O2—Zn1	101.8 (3)	C17—C18—H18B	109.5
C14—O3—Zn1	79.6 (3)	H18A—C18—H18B	109.5
C1—O4—Zn1^i^	97.0 (3)	C17—C18—H18C	109.5
C1—O5—Zn1^i^	85.9 (4)	H18A—C18—H18C	109.5
H1WA—O1W—H1WB	107.7	H18B—C18—H18C	109.5
O5—C1—O4	119.3 (5)	C20—C19—C17	120.1 (3)
O5—C1—C2	120.5 (5)	C20—C19—H19	119.9
O4—C1—C2	120.1 (4)	C17—C19—H19	119.9
O5—C1—Zn1^i^	65.3 (3)	N1—C20—C19	121.8 (3)
O4—C1—Zn1^i^	54.0 (3)	N1—C20—C21	115.6 (3)
C2—C1—Zn1^i^	171.7 (4)	C19—C20—C21	122.7 (3)
C7—C2—C3	117.3 (4)	N2—C21—C22	121.3 (3)
C7—C2—C1	122.8 (4)	N2—C21—C20	115.0 (3)
C3—C2—C1	119.6 (4)	C22—C21—C20	123.7 (3)
C4—C3—C2	122.0 (4)	C21—C22—C23	120.4 (3)
C4—C3—H3	119.0	C21—C22—H22	119.8
C2—C3—H3	119.0	C23—C22—H22	119.8
C5—C4—C3	119.6 (4)	C22—C23—C25	117.8 (3)
C5—C4—H4	120.2	C22—C23—C24	120.9 (3)
C3—C4—H4	120.2	C25—C23—C24	121.4 (4)
C4—C5—C6	120.4 (4)	C23—C24—H24A	109.5
C4—C5—H5	119.8	C23—C24—H24B	109.5
C6—C5—H5	119.8	H24A—C24—H24B	109.5
C5—C6—C7	119.6 (4)	C23—C24—H24C	109.5
C5—C6—H6	120.2	H24A—C24—H24C	109.5
C7—C6—H6	120.2	H24B—C24—H24C	109.5
C6—C7—O1	115.8 (3)	C26—C25—C23	119.2 (4)
C6—C7—C2	121.0 (4)	C26—C25—H25	120.4
O1—C7—C2	123.0 (3)	C23—C25—H25	120.4
O1—C8—C9	121.5 (3)	N2—C26—C25	122.9 (3)
O1—C8—C13	118.0 (3)	N2—C26—H26	118.5
C9—C8—C13	120.3 (3)	C25—C26—H26	118.5
			
O2—Zn1—N1—C15	−82.0 (3)	C1—C2—C7—C6	−172.7 (4)
O4^i^—Zn1—N1—C15	95.9 (4)	C3—C2—C7—O1	176.2 (3)
N2—Zn1—N1—C15	179.7 (3)	C1—C2—C7—O1	1.9 (6)
O5^i^—Zn1—N1—C15	72.7 (3)	C7—O1—C8—C9	14.3 (6)
O3—Zn1—N1—C15	−25.3 (3)	C7—O1—C8—C13	−169.4 (3)
C1^i^—Zn1—N1—C15	79.1 (4)	O1—C8—C9—C10	175.9 (4)
O2—Zn1—N1—C20	90.9 (3)	C13—C8—C9—C10	−0.3 (6)
O4^i^—Zn1—N1—C20	−91.2 (4)	C8—C9—C10—C11	−1.5 (7)
N2—Zn1—N1—C20	−7.4 (2)	C9—C10—C11—C12	1.7 (7)
O5^i^—Zn1—N1—C20	−114.4 (3)	C10—C11—C12—C13	−0.1 (7)
O3—Zn1—N1—C20	147.7 (3)	C11—C12—C13—C8	−1.6 (6)
C1^i^—Zn1—N1—C20	−107.9 (3)	C11—C12—C13—C14	176.4 (4)
O2—Zn1—N2—C26	78.6 (3)	O1—C8—C13—C12	−174.5 (3)
O4^i^—Zn1—N2—C26	−23.5 (4)	C9—C8—C13—C12	1.8 (6)
N1—Zn1—N2—C26	−176.9 (3)	O1—C8—C13—C14	7.6 (6)
O5^i^—Zn1—N2—C26	−81.7 (3)	C9—C8—C13—C14	−176.1 (4)
O3—Zn1—N2—C26	105.1 (4)	Zn1—O3—C14—O2	6.0 (4)
C1^i^—Zn1—N2—C26	−51.9 (4)	Zn1—O3—C14—C13	−173.3 (3)
O2—Zn1—N2—C21	−98.6 (3)	Zn1—O2—C14—O3	−7.5 (5)
O4^i^—Zn1—N2—C21	159.4 (3)	Zn1—O2—C14—C13	171.8 (3)
N1—Zn1—N2—C21	5.9 (3)	C12—C13—C14—O3	11.0 (6)
O5^i^—Zn1—N2—C21	101.1 (3)	C8—C13—C14—O3	−171.1 (4)
O3—Zn1—N2—C21	−72.0 (4)	C12—C13—C14—O2	−168.3 (4)
C1^i^—Zn1—N2—C21	130.9 (3)	C8—C13—C14—O2	9.6 (6)
O4^i^—Zn1—O2—C14	−90.3 (3)	C20—N1—C15—C16	−1.4 (6)
N2—Zn1—O2—C14	170.5 (2)	Zn1—N1—C15—C16	171.3 (3)
N1—Zn1—O2—C14	88.7 (3)	N1—C15—C16—C17	−0.6 (6)
O5^i^—Zn1—O2—C14	−43.6 (4)	C15—C16—C17—C19	1.6 (6)
O3—Zn1—O2—C14	3.8 (2)	C15—C16—C17—C18	−177.8 (4)
C1^i^—Zn1—O2—C14	−72.9 (3)	C16—C17—C19—C20	−0.7 (6)
O2—Zn1—O3—C14	−3.8 (2)	C18—C17—C19—C20	178.8 (4)
O4^i^—Zn1—O3—C14	92.7 (3)	C15—N1—C20—C19	2.4 (5)
N2—Zn1—O3—C14	−35.5 (4)	Zn1—N1—C20—C19	−171.3 (3)
N1—Zn1—O3—C14	−110.0 (2)	C15—N1—C20—C21	−178.6 (3)
O5^i^—Zn1—O3—C14	151.1 (2)	Zn1—N1—C20—C21	7.8 (4)
C1^i^—Zn1—O3—C14	122.1 (3)	C17—C19—C20—N1	−1.4 (6)
Zn1^i^—O5—C1—O4	1.9 (5)	C17—C19—C20—C21	179.6 (3)
Zn1^i^—O5—C1—C2	−173.3 (4)	C26—N2—C21—C22	−0.5 (5)
Zn1^i^—O4—C1—O5	−2.1 (5)	Zn1—N2—C21—C22	176.9 (3)
Zn1^i^—O4—C1—C2	173.1 (3)	C26—N2—C21—C20	179.0 (3)
O5—C1—C2—C7	−172.9 (5)	Zn1—N2—C21—C20	−3.6 (4)
O4—C1—C2—C7	12.0 (7)	N1—C20—C21—N2	−2.9 (5)
O5—C1—C2—C3	12.9 (7)	C19—C20—C21—N2	176.2 (3)
O4—C1—C2—C3	−162.2 (4)	N1—C20—C21—C22	176.6 (3)
C7—C2—C3—C4	−1.7 (7)	C19—C20—C21—C22	−4.3 (6)
C1—C2—C3—C4	172.8 (4)	N2—C21—C22—C23	−0.6 (6)
C2—C3—C4—C5	0.1 (7)	C20—C21—C22—C23	179.9 (3)
C3—C4—C5—C6	1.8 (7)	C21—C22—C23—C25	1.0 (6)
C4—C5—C6—C7	−1.9 (7)	C21—C22—C23—C24	−179.8 (4)
C5—C6—C7—O1	−174.8 (4)	C22—C23—C25—C26	−0.3 (6)
C5—C6—C7—C2	0.2 (6)	C24—C23—C25—C26	−179.5 (4)
C8—O1—C7—C6	−113.1 (4)	C21—N2—C26—C25	1.2 (6)
C8—O1—C7—C2	72.0 (5)	Zn1—N2—C26—C25	−175.9 (3)
C3—C2—C7—C6	1.6 (6)	C23—C25—C26—N2	−0.8 (6)

Symmetry codes: (i) −*x*+1, −*y*+2, −*z*+1.

### Hydrogen-bond geometry (Å, °)

**Table:**

*D*—H···*A*	*D*—H	H···*A*	*D*···*A*	*D*—H···*A*
O1W—H1WA···O3	0.85	2.04	2.845 (5)	158
O1W—H1WB···O5^i^	0.85	2.07	2.876 (6)	158

Symmetry codes: (i) −*x*+1, −*y*+2, −*z*+1.
